# Significant association between lncRNA H19 polymorphisms and cancer susceptibility: a meta-analysis

**DOI:** 10.18632/oncotarget.16658

**Published:** 2017-03-29

**Authors:** Xue-Feng Li, Xin-Hai Yin, Jun-Wei Cai, Ming-Ju Wang, Yu-Qin Zeng, Min Li, Yu-Ming Niu, Ming Shen

**Affiliations:** ^1^ Department of Endocrinology, Taihe Hospital, Hubei University of Medicine, Shiyan 442000, China; ^2^ Department of Oral and Maxillary Surgery, Gui Zhou Provincial People's Hospital, Guiyang 550000, China; ^3^ Information Resources, Taihe Hospital, Hubei University of Medicine, Shiyan 442000, China; ^4^ Department of Stomatology and Center for Evidence-Based Medicine and Clinical Research, Taihe Hospital, Hubei University of Medicine, Shiyan 442000, China; ^5^ Jiangsu Key Laboratory of Oral Diseases, Department of Dental Implant, Affiliated Hospital of Stomatology, Nanjing Medical University, Nanjing 210029, China

**Keywords:** H19, polymorphism, cancer, meta-analysis

## Abstract

Previous epidemiological research suggests polymorphisms in long non-coding RNA (lncRNA) H19 are associated with an increased risk of cancer, but the results are inconsistent. We therefore conducted a meta-analysis to more accurately determine the association between lncRNA H19 polymorphisms and cancer risk. The PubMed, Embase, and Science Citation Index online databases were searched and 11 relevant studies involving a total of 33,209 participants were identified. Odds ratios (ORs) and corresponding 95% confidence interval (CIs) from these studies were used to detect associations between H19 polymorphisms and cancer risk using five genetic models. The pooled result suggested that the rs2839698 G>A polymorphism was associated with digestive cancer risk in all five models. Moreover, a protective effect against cancer development was observed for the T allele variant of the rs2107425 C>T polymorphism, especially in Caucasian patient populations. No significant associations were found between lncRNA H19 rs217727 G>A polymorphism and cancer risk. In summary, the rs2839698 G>A and rs2107425 C>T polymorphisms in lncRNA H19 may therefore play opposing roles during cancer development, and their effects may vary depending on cancer type and patient ethnicity.

## INTRODUCTION

Cancer is a leading cause of death worldwide and has major economic impacts in both developed and developing countries [1, 2]. In 2012, approximately 14.1 million patients were diagnosed with cancer, and 8.2 million people died due to cancer, according to a GLOBOCAN reported [3]. Longer life expectancy, an aging population, and increased environmental pollution may all contribute to increasing incidences of cancer in China and other countries [4]. Other risk factors, such as unhealthy dietary practices and lifestyles, chronic inflammation, and viral infections, also contribute to the development of cancer [5–9]. However, the mechanisms underlying cancer pathogenesis remain largely unknown.

Increasing evidence indicates that abnormal gene regulation and expression result in abnormal protein activity and disruption of the cell cycle, in turn leading to tumor formation. Long non-coding RNAs (lncRNAs), which were first identified in the 1990s, are single-stranded, non-coding RNAs with lengths of more than 200 nucleotides and no open reading frames [10]. Molecular studies have shown that lncRNAs play important roles in cell cycle regulation and affect proliferation, differentiation, and apoptosis [11]. LncRNAs are also important regulators of tissue pathology and disease processes related to cancer [12].

Thousands of lncRNA molecules have been identified to date in the human genome. The lncRNA H19 is located on chromosome 11p15.5 and is 2.3 kb in length [13]. The H19 gene, which is a paternally imprinted onco-fetal gene, is typically down-regulated in adult tissues, but can be expressed in cancer tissues at levels similar to those observed in fetal life [14]. Many studies have confirmed that H19 is re-expressed in many types of solid tumors, such as breast cancer, gastric cancer, and esophageal cancer, and H19 expression is closely related to tumor invasion, metastasis, recurrence and poor prognosis [15, 16].

Genetic mutations, such as single nucleotide polymorphisms (SNPs), also influence susceptibility to cancer [17]. Previous studies have also identified associations between cancer risk and SNPs located in lncRNAs; examples include the HOTAIR rs920778 C>T polymorphism in esophageal cancer and the PRNCR1 rs13252298 A>G polymorphism in gastric cancer [18, 19]. Associations have also been reported between the three most common SNPs in H19 (rs2839698 G>A, and rs217727 G>A, rs2107425 C>T) and cancer susceptibility. In 2008, Verhaegh et al. conducted the first case-control study and found that the heterozygote H19 rs2839698 G>A polymorphism might be associated with bladder cancer risk in a Caucasian population [20]. Subsequent studies investigating the association between the lncRNA H19 polymorphisms and cancer susceptibility have reported inconsistent results. Therefore, in this meta-analysis, we examined the association between lncRNA H19 polymorphisms and cancer susceptibility in all relevant published studies. Our meta-analysis was preformed according to the Preferred Reporting Items for Systematic Reviews and Meta-Analyses (PRISMA) statement [21]. No ethical issues were involved in this study given that our data were based on published studies.

## RESULTS

### Study characteristics

**Figure 1:**
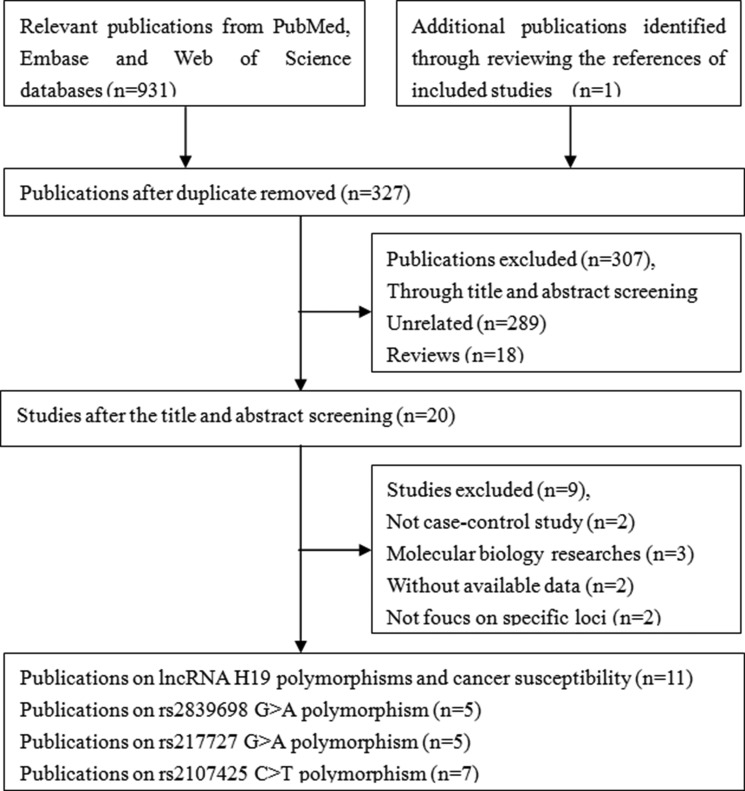
Flow diagram of the study selection process

A systematic literature search identified 932 potentially relevant articles. At the end of the gradual selection process, eleven published articles involving a total of 14,030 cancer patients and 19,179 healthy controls met our inclusion criteria and were included in this meta-analysis (Figure 1) [20, 22–31]. Among the eleven eligible articles, five studies examined Asian populations, seven examined Caucasian populations and one examined an African populations (one article included two different races, Caucasian and African population). The characteristics of the included studies are presented in Table 1.

**Table 1:** Characteristics of included studies on lncRNA H19 polymorphisms and cancer risk included in the meta-analysis

First author	Year	Country/Region	Racial	Source of controls	Case	Control	Genotype distribution						Genotyping methods	*P* for HWE^a^	MAF in control	Type	NOS
							Case			Control							
							H19 rs2839698 G>A										
							GG	GA	AA	GG	GA	AA					
Verhaegh	2008	Netherlands	Caucasian	PB	177	204	54	74	49	52	109	43	PCR-RFLP	0.31	0.48	Bladder	8
Yang	2015	China	Asian	HB	500	500	250	195	55	284	178	38	TaqMan	0.18	0.25	Gastric	8
Li	2016	China	Asian	PB	1147	1203	583	462	102	666	462	75	TaqMan	0.67	0.25	Colorectal	9
Hua	2016	China	Asian	HB	1049	1397	552	418	79	729	565	103	TaqMan	0.65	0.28	Bladder	9
Gong	2016	China	Asian	HB	496	206	237	220	39	99	80	27	TaqMan	0.10	0.33	Lung	8
							**H19 rs217727 G>A**										
							**GG**	**GA**	**AA**	**GG**	**GA**	**AA**					
Verhaegh	2008	Netherlands	Caucasian	PB	177	204	114	59	4	115	80	9	PCR-RFLP	0.29	0.24	Bladder	8
Yang	2015	China	Asian	HB	500	500	160	252	88	193	244	63	TaqMan	0.30	0.37	Gastric	8
Li	2016	China	Asian	PB	1147	1203	480	514	153	456	570	177	TaqMan	0.96	0.38	Colorectal	9
Hua	2016	China	Asian	HB	1046	1394	431	467	148	573	665	156	TaqMan	0.07	0.35	Bladder	9
Xia	2016	China	Asian	PB	464	467	160	156	148	139	212	116	CRS-RFLP	0.05	0.48	Breast	10
							**H19 rs2107425 C>T**										
							**CC**	**CT**	**TT**	**CC**	**CT**	**TT**					
Verhaegh	2008	Netherlands	Caucasian	PB	177	204	92	65	20	89	96	19	PCR-RFLP	0.34	0.33	Bladder	8
Bhatti	2008	USA	Caucasian	PB	824	1073	392	432		502	571		Sequenom	NA	NA	Breast	6
Quay	2009	Mixed	Caucasian	PB	1460	2463	767	544	149	1118	1098	247	TaqMan	0.34	0.32	Ovarian	8
Song	2009	Mixed	Caucasian	PB	5366	8538	2619	2192	555	4029	3667	842	TaqMan	0.86	0.31	Ovarian	9
Butt	2012	Sweden	Caucasian	PB	678	1355	360	250	68	637	573	145	MassArray	0.34	0.32	Breast	8
Barnholtz-Sloan11	2014	USA	Caucasian	PB	1225	1118	604	516	105	521	478	119	Illumina	0.55	0.32	Breast	10
Barnholtz-Sloan12	2014	USA	Africa	PB	737	658	161	390	186	170	339	149	Illumina	0.42	0.48	Breast	10
Gong	2016	China	Asian	HB	479	203	181	235	63	79	96	28	TaqMan	0.89	0.37	Lung	8

^a^HWE in control

MAF: Minor allele frequency in control group.

PB: Population-based HB: Hospital-based.

CRS-RFLP: Created restriction site-restriction fragment length polymorphism.

### Meta-analysis of the lncRNA H19 rs2839698 G>A polymorphism and cancer risk

The association between the H19 rs2839698 G>A polymorphism and cancer risk was examined in five relevant studies involving 3,369 patients and 3,510 healthy controls. No significant associations were identified between overall cancer risk and this SNP in any of the five genetic models. However, subgroup analyses by cancer type indicated that rs2839698 G>A was associated with an increase indigestive cancer risk (A vs. G: OR = 1.23, 95% CI = 1.10–1.36, *P* < 0.01, *I*^2^ = 0%; GA vs. GG: OR = 1.17, 95% CI = 1.02–1.35, *P* = 0.03, *I*^2^ = 0%; AA vs. GG: OR = 1.58, 95% CI = 1.22–2.05, *P* < 0.01, *I*^2^ = 0%; GA+AA vs. GG: OR = 1.23, 95% CI = 1.08–1.41, *P* < 0.01, *I*^2^ = 0%; AA vs. GG+GA: OR = 1.48, 95% CI = 1.15–1.90, *P* < 0.01, *I*^2^ = 0%) (Table 2). Additional associations were identified in the other subgroup analysis (Table 2, Supplementary Figure 1). Furthermore, meta-regression analyses did not reveal any critical factors to explain these heterogeneities, while stratified analyses by cancer type relieved the heterogeneity.

**Table 2:** Summary ORs and 95% CI of lncRNA H19 polymorphisms and cancer risk

Locus	N*	No. of case/control	OR	95% CI	*P*	*I*^2^ (%)^a^		OR	95% CI	*P*	*I*^2^ (%)^a^		OR	95% CI	*P*	*I*^2^ (%)^a^		OR	95% CI	*P*	*I*^2^ (%)^a^		OR	95% CI	*P*	*I*^2^ (%)^a^
rs2839698 G>A			**A vs. G**					**GA vs. GG**					**AA vs. GG**					**GA+AA vs. GG**					**AA vs. GG+GA**			
Total	5	3369/3510	1.08	0.96-1.23	0.21	58.8		1.06	0.91-1.23	0.44	43.8		1.15	0.83-1.58	0.42	66.1		1.08	0.94-1.25	0.27	46.5		1.15	1.85-1.57	0.36	67.4
Ethnicity																										
Asian	4	3192/3306	1.09	0.94-1.27	0.24	68.6		1.09	0.98-1.21	0.10	0		1.15	0.78-1.69	0.48	74.4		1.12	0.97-1.28	0.11	42.3		1.10	0.76-1.59	0.63	74.0
Design																										
PB	2	1324/1407	1.17	1.04-1.31	0.01	0		0.91	0.53-1.55	0.72	78.2		1.43	1.08-1.88	0.01	10.9		1.02	0.68-1.54	0.93	68.2		1.46	1.13-1.89	<0.01	0
HB	3	2045/2103	1.05	0.86-1.28	0.63	69.8		1.08	0.92-1.26	0.36	20.6		1.02	0.62-1.67	0.93	74.7		1.08	0.90-1.31	0.40	47.6		0.98	0.61-1.57	0.92	75.0
Type																										
Bladder cancer	2	1226/1601	1.00	0.89-1.12	1.00	0		0.85	0.59-1.24	0.41	58.0		1.03	0.79-1.36	0.82	0		0.96	0.82-1.11	0.57	0		1.13	0.88-1.46	0.35	28.0
Digestive cancer	2	1647/1703	1.23	1.10-1.36	<0.01	0		1.17	1.02-1.35	0.03	0		1.58	1.22-2.05	<0.01	0		1.23	1.08-1.41	<0.01	0		1.48	1.15-1.90	<0.01	0
																										
rs217727 G>A			**A vs. G**					**GA vs. GG**					**AA vs. GG**					**GA+AA vs. GG**					**AA vs. GG+GA**			
Total	5	3334/3768	1.01	0.88-1.07	0.86	72.6		0.88	0.73-1.06	0.18	65.0		0.60	0.26-1.39	0.23	16.0		0.94	0.78-1.12	0.47	66.3		1.19	0.93-1.53	0.16	64.9
Ethnicity																										
Asian	4	3157/3564	1.05	0.91-1.21	0.49	73.6		0.90	0.73-1.11	0.32	71.9		0.50	0.20-1.25	0.14	23.3		0.97	0.80-1.17	0.76	70.3		1.23	0.97-1.57	0.09	68.0
Design																										
PB	3	1788/1874	0.92	0.84-1.01	0.09	46.6		0.79	0.69-0.91	<0.01	26.6		1.07	0.36-3.22	0.90	0		0.82	0.72-0.94	<0.01	0		1.03	0.66-1.58	0.91	73.5
HB	2	1546/1894	1.15	0.97-1.37	0.12	62.9		1.06	0.80-1.40	0.70	67.3		0.26	0.06-1.20	0.08	31.5		1.13	0.85-1.51	0.39	71.6		1.36	1.12-1.66	<0.01	0
Type																										
Bladder cancer	2	1223/1598	0.92	0.65-1.31	0.65	73.4		0.90	0.77-1.06	0.22	0		0.51	0.04-6.77	0.61	62.5		0.89	0.66-1.21	0.47	53.5		0.97	0.41-2.32	0.95	58.0
Digestive cancer	2	1647/1703	1.06	0.75-1.50	0.75	90.5		1.02	0.71-1.47	0.93	80.4		0.52	0.10-2.81	0.45	0		1.05	0.67-1.64	0.82	88.0		1.13	0.69-1.85	0.63	82.1
																										
rs2107425 C>T			T vs. C					CT vs. CC					TT vs. CC					CT+TT vs. CC					TT vs. CC+CT			
Total	8	10946/15612	0.94	0.86-1.01	0.09	60.8		0.88	0.77-1.00	0.05	70.4		0.97	0.89-1.06	0.51	32.4		0.87	0.78-0.97	0.02	68.4		1.02	0.94-1.11	0.60	0
Ethnicity																										
Caucasian	6	9730/14751	0.90	0.84-0.97	0.01	51.8		0.82	0.72-0.94	<0.01	69.1		0.94	0.86-1.04	0.22	13.9		0.83	0.75-0.92	<0.01	62.9		1.01	0.92-1.10	0.85	8.1
Design																										
PB	7	10467/15409	0.93	0.85-1.01	0.09	66.7		0.86	0.75-0.99	0.03	73.9		0.95	0.83-1.10	0.50	43.6		0.86	0.77-0.97	0.01	71.7		1.02	0.94-1.11	0.57	6.5
Type																										
breast cancer	4	3464/4204	0.95	0.80-1.13	0.57	77.7		0.94	0.75-1.19	0.62	73.2%		0.94	0.67-1.31	0.72	73.0		0.89	0.74-1.08	0.24	73.3		0.95	0.76-1.20	0.68	51.8
Ovarian cancer	2	6826/11001	0.92	0.80-1.05	0.20	82.5		0.82	0.65-1.05	0.10	89.2		0.98	0.89-1.09	0.74	18.4		0.84	0.68-1.05	0.13	88.6		1.05	0.95-1.16	0.37	0.0

^*^ Numbers of comparisons.

^a^ Test for heterogeneity.

PB: Population-based HB: Hospital-based.

RT-PCR: Real-time PCR

The sensitivity analysis, which was conducted by omitting studies one by one to examine the stability of the pooled ORs, revealed a slight change when data from Li et al.'s study was removed [29] (Supplementary Figure 2). Cumulative analysis by publication date also indicated that the observed dangerous association increased in strength when the same study, which was published in 2016, was included [29] (Supplementary Figure 3).

Potential publication bias was evaluated using Egger's linear regression and Begg's funnel plots. No significant asymmetrical funnel plots were observed, which were guaranteed by the Egger's test (A vs. G: *P* = 0.77; GA vs. GG: *P* = 0.62; AA vs. GG: *P* = 0.52; GA+AA vs. GG: *P* = 0.62; AA vs. GG+GA: *P* = 0.60) (Supplementary Figure 4).

### Meta-analysis of the lncRNA H19 rs217727 G>A polymorphism and cancer risk

The association between lncRNA H19 rs217727 G>A polymorphism and cancer risk was examined in five studies involving 3,334 cases and 3,768 controls. No significant overall associations were found in any of the five genetic models (A vs. G: OR = 1.01, 95% CI = 0.88–1.07, *P* = 0.86, *I*^2^ = 72.6%; GA vs. GG: OR = 0.88, 95% CI= 0.73–1.06, *P* = 0.18, *I*^2^ = 65.0%; AA vs. GG: OR = 0.60, 95% CI = 0.26–1.39, *P* = 0.23, *I*^2^ = 16.0%; GA+AA vs. GG: OR = 0.94, 95% CI = 0.78–1.12, *P* = 0.47, *I*^2^ = 66.3%; AA vs. GG+GA: OR = 1.19, 95% CI = 0.93–1.53, *P* = 0.16, *I*^2^ = 64.9%) (Table 2, Supplementary Figure 5). In contrast, two genetic models (GA vs. GG: OR = 0.79, 95% CI = 0.69–0.91, *P* < 0.01, *I*^2^ = 26.6%; GA+AA vs. GG: OR = 0.82, 95% CI = 0.72–0.94, *P* < 0.01, *I*^2^ = 0%) indicated protective effects on cancer risk in population-base subgroup analysis. Additional subgroup analyses based on ethnicity and cancer type did not identify any significant associations. Meta-regression analyses and stratified analyses were also conducted, but no critical factors were found that explain the observed heterogeneities.

No substantial changes were observed in the sensitivity and cumulative analyses (Supplementary Figures 6 and 7), and no publication bias was observed (A vs. G: *P* = 0.91; GA vs. GG: *P* = 0.70; AA vs. GG: *P* = 0.90; GA+AA vs. GG: *P* = 0.86; AA vs. GG+GA: *P* = 0.79) (Supplementary Figure 8).

### Meta-analysis of the lncRNA H19 rs2107425 C>T polymorphism and cancer risk

**Figure 2:**
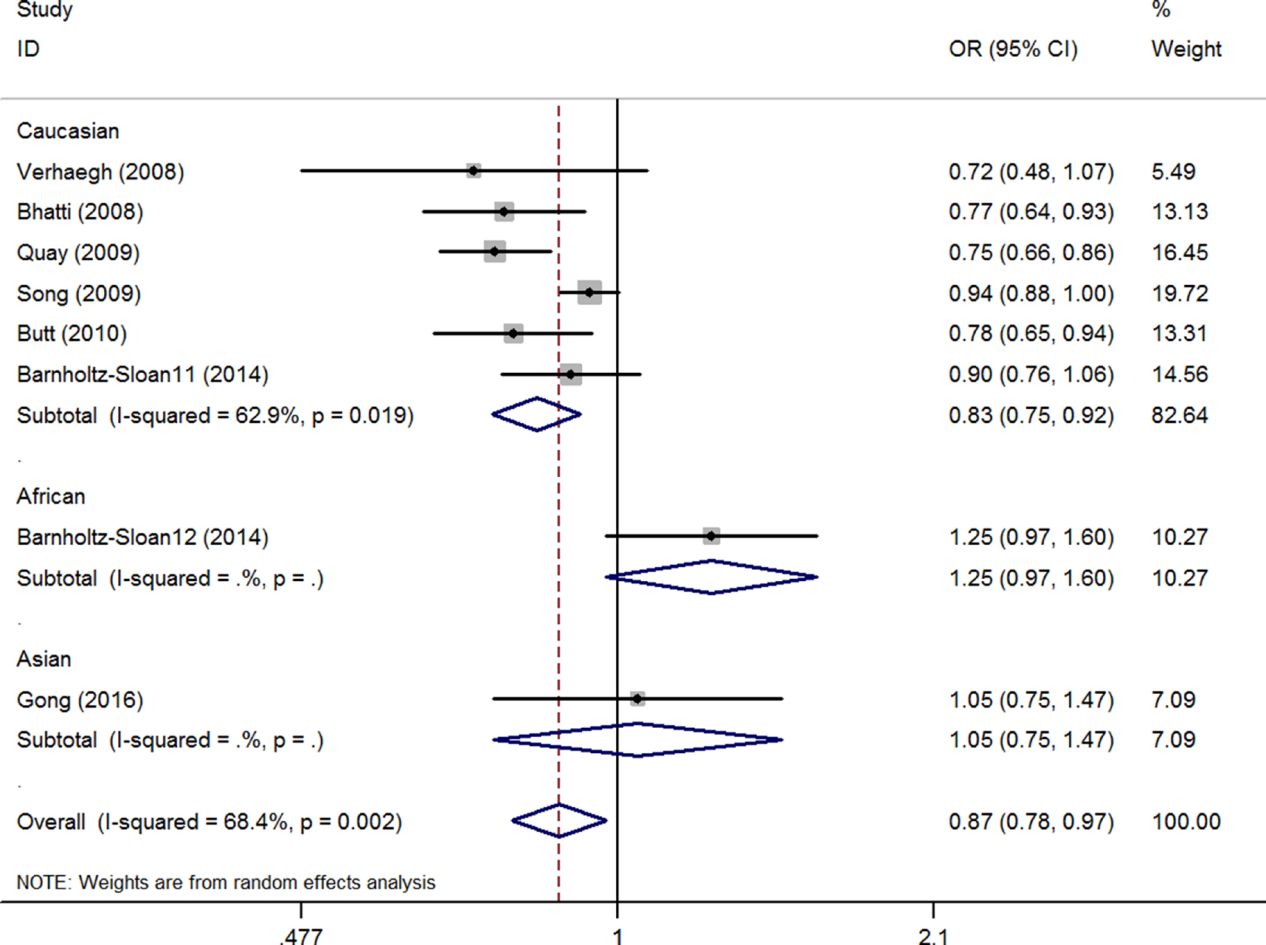
OR and 95% CIs of the associations between lncRNA H19 rs2107425 C>T polymorphism and cancer risk in CT+TT vs. CC model

The association between the H19 rs2107425 C>T polymorphism and cancer risk was examined in eight studies involving 10,856 patients and 15,612 healthy controls. A protective effect on cancer development was observed in the variant T allele (CT vs. CC: OR = 0.88, 95% CI = 0.77–1.00, *P* = 0.05, *I*^2^ = 70.4%; CT+TT vs. CC: OR = 0.87; 95% CI = 0.78–0.97, *P* = 0.02). The same association with decreased risk was also observed in Caucasian populations in the subgroup analyses by race (T vs. C: OR = 0.90, 95% CI = 0.84–0.97, *P* = 0.01, *I*^2^ = 51.8%; CT vs. CC: OR = 0.82, 95% CI = 0.72–0.94, *P* = < 0.01, *I*^2^ = 69.1%; CT+TT vs. CC: OR = 0.83, 95% CI = 0.75–0.92, *P* < 0.01, *I*^2^ = 62.9%, (Figure 2, Supplementary Figure 9) (Table 2). Meta-regression analyses indicated that ethnicity explained the Ʈ2 values in these genetic models (T vs. C: 58.7%, *P* = 0.06; CT vs. CC: 54.9%, *P* = 0.06; CT+TT vs. CC: 59.8%, *P* = 0.03).

**Figure 3:**
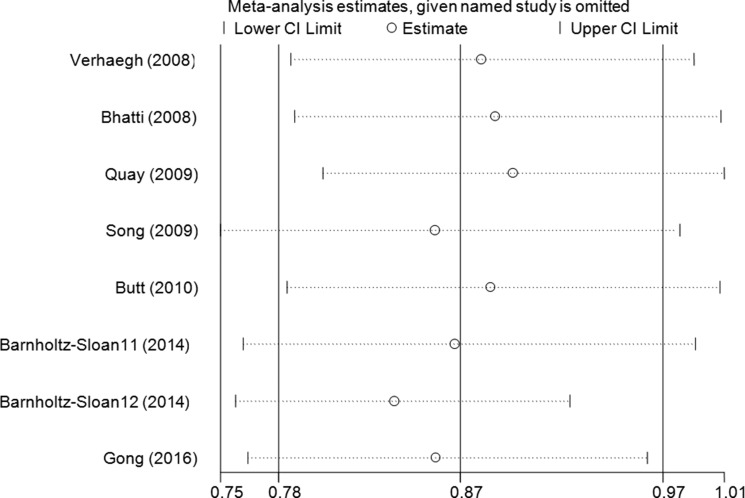
Sensitivity analysis through deleting each study to reflect the influence of the individual dataset to the pooled ORs between lncRNA H19 rs2107425 C>T polymorphism and cancer risk in CT+TT vs. CC model

**Figure 4:**
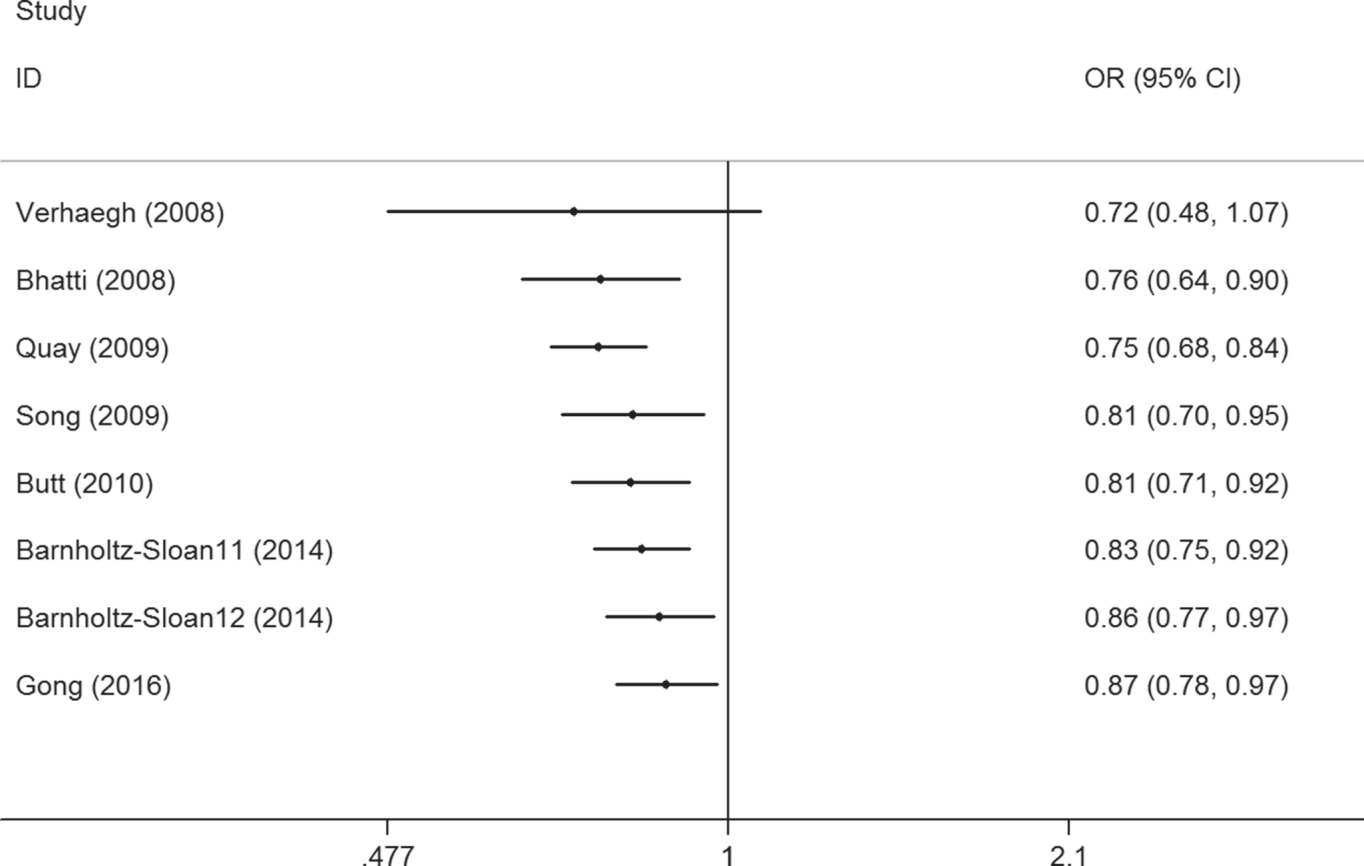
Cumulative meta-analyses according to publication year between lncRNA H19 rs2107425 C>T polymorphism and cancer risk in CT+TT vs. CC model

Sensitivity analysis was conducted by omitting studies one at a time to see whether the pooled ORs were altered, and a slight bias was found when the data from Barnholtz-Sloan et al.'s study in an African patient population was removed (Figure 3, Supplementary Figure 10). No changes were observed in the cumulative analysis by publication date (Figure 4, Supplementary Figure 11).

**Figure 5:**
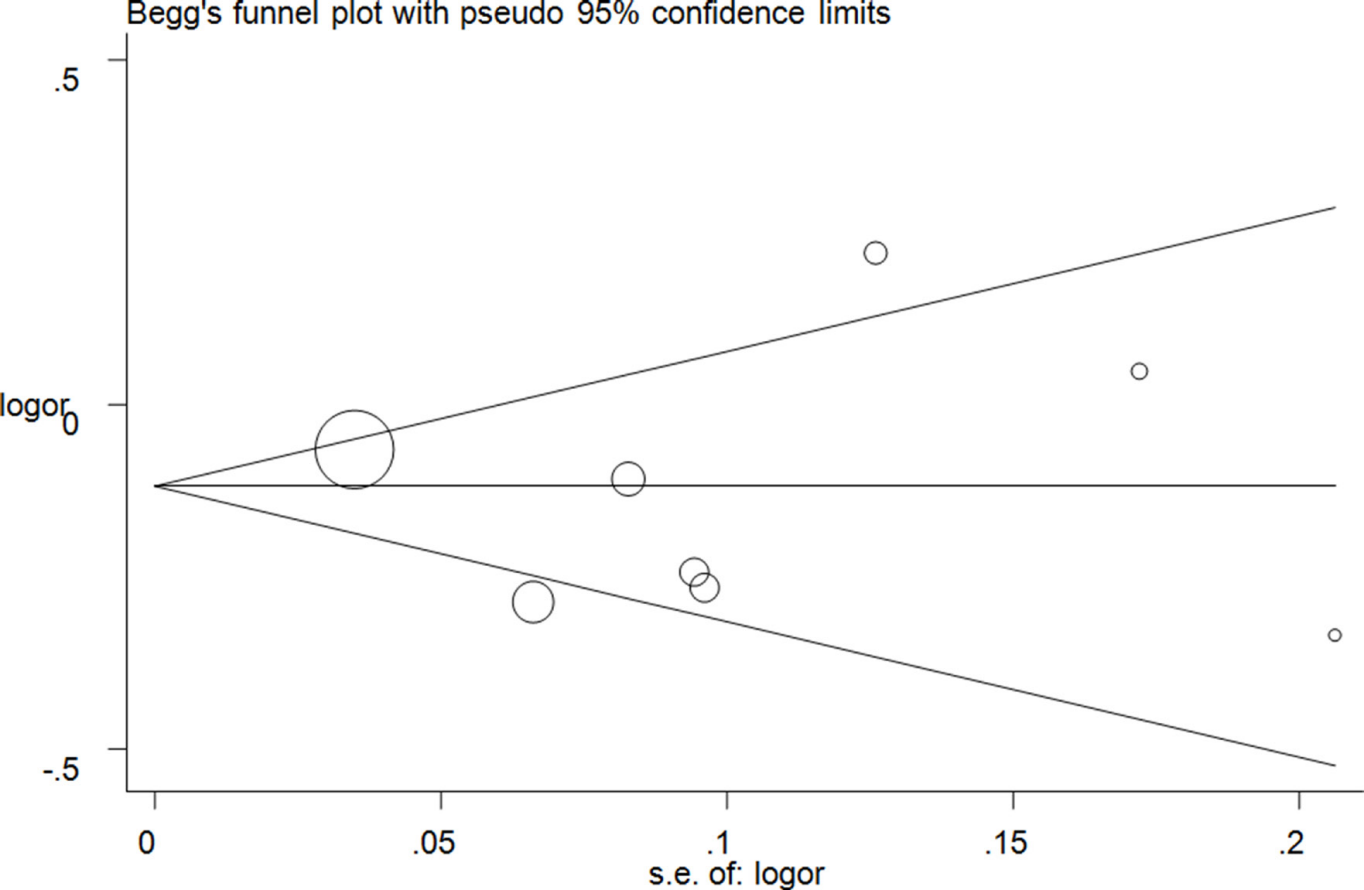
Funnel plot analysis of the publication bias of lncRNA H19 rs2107425 C>T polymorphism and cancer risk in CT+TT vs. CC model. Circles represent the weight of the studies

Publication bias was evaluated using Egger's linear regression and Begg's funnel plots. No significant asymmetrical funnel plots were observed, which were guaranteed by the Egger's test (T vs. C: *P* = 0.72; CT vs. CC: *P* = 0.88; TT vs. CC: *P* = 0.72, (Figure 5); CT+TT vs. CC: *P* = 0.71; TT vs. CC+CT: *P* = 0.59) (Supplementary Figure 12).

## DISCUSSION

Cancer is currently a main cause of poverty and death worldwide. However, the fundamental mechanism of the pathogenesis of cancer remains unclear. Noncoding RNAs, including lncRNAs, affect many cellular processes by regulating gene function and maintaining cellular homeostasis, both of which are closely related to organ differentiation and disease development [32, 33]. Abnormal expression of lncRNAs is closely linked to the occurrence, development and postoperative recovery from cancer; thus, lncRNAs are likely to be useful diagnostic and prognostic markers and might also serve as targets for lncRNA-mediated therapies [34].

Large numbers of SNP loci have been identified in many lncRNAs, and an increasing number of studies have focused on the association between lncRNA polymorphisms and cancer risk. LncRNAs are approximately 200 nucleotide long RNA molecules that are stably expressed in plasma but do not code for proteins. Accumulating evidence has demonstrated that abnormal expressions of lncRNAs are often promotes tumorigenesis through by interfering with normal cell cycle progression [35]. The H19 gene belong to a highly conserved imprinted gene that affects embryonic development and growth [36]. Recent studies have demonstrated that lncRNA H19 plays important roles in carcinogenesis and cancer metastasis. For example, Luo et al found that up-regulated H19 enhances bladder cancer metastasis with EZH2 and inhibiting E-cad expression [16]. In addition, Han et al also found that increased H19 expression was associated with tumor grade and TNM stage in colorectal cancer patients [37]. Furthermore, growing evidences suggests that polymorphisms in the H19 gene affect cancer development. In 2008, H19 polymorphisms were first examined by Verhaegh et al. [20], and a series of case-control studies have since been conducted. However, additional studies are needed to confirm those results due to the limited number of studies and small sample sizes involved.

Meta-analysis can be used to integrate data from multiple studies, thereby expanding sample sizes and increasing the strength of conclusions [38]. We conducted this meta-analysis of eleven studies to more accurately assess the associations between the lncRNA H19 polymorphisms rs2839698 G>A, rs217727 G>A and rs2107425 C>T polymorphisms and cancer risk. Our results revealed that the lncRNA H19 rs2839698 G>A polymorphism might be an important risk factor for developing gastric cancer and colorectal cancer. In contrast, the T allele variant of the lncRNA H19 rs2107425 C>T polymorphism had a protective effect against cancer development, especially in Caucasian population. Stratified analyses suggested that differences in cancer location and patient ethnicity might have contributed to discrepancies among the studies examined. The lack of significant deviations in the sensitivity and publication bias analyses indicated that the pooled results were stable and credible. In 2016, Lu et al. conducted the first meta-analysis of the association between a singleH19 polymorphism locus (rs217727) and cancer risk in four studies. Their results indicated that this polymorphism was not associated with overall cancer risk [39]. In addition, Chu et al. carried out another meta-analysis assessing the association between H19 rs2839698 G>A, rs217727 G>A and rs2107425 C>T polymorphisms and cancer risk through aggregating ten published articles [40]. They suggested that the rs2107425 C>T polymorphism correlated with a significantly decreased risk but the rs2839698 G>A polymorphism exhibited a significantly higher risk of developing cancer. Compared with the previous meta-analysis, our meta-analysis used more scientific retrieval strategy and our meta-analysis collected more research subjects (eleven published studies involving 33,209 participants). Furthermore, our meta-analysis performed the quality evaluation with modified Newcastle-Ottawa scale (NOS), which could make a correct evaluation of the quality of the included studies. In addition, meta-regression was also conducted to explore the causes of heterogeneity during our analysis.

To the best of our knowledge, this is the first meta-analysis to examine the association between these three lncRNA H19 polymorphisms and the risk of different types of cancer. Although only eleven studies involving relatively small sample sizes of 14,030 cases and 19,179 controls were included, these results help to characterize associations between lncRNA H19 polymorphisms and cancer risk. Our conclusions are further supported by the observation that genotype distributions of the selected SNP loci in the healthy controls were consistent with HWE and by the lack of any significant publication bias. However, due to the relatively small number of studies included and to limitations of the linear regression method employed, additional studies should be conducted to confirm these results and reduce the effects of possible publication biases.

Some additional limitations of this meta-analysis should be considered when interpreting the results. First, only eleven studies involving the three SNP loci examined were identified; the small number sizes might decrease the reliability of the results and increases the probability of random errors, thus affecting the assessment of associations between these lncRNA H19 polymorphisms and cancer susceptibility. Second, some important patient characteristics, such as cigarette use and alcohol consumption, as well as other environmental factors, were not considered. The impact of interactions between genetic and environmental factors on cancer development could not be assessed in this study. Third, all of the included studies focused almost exclusively on Asian or Caucasian populations, and our results may not be applicable to all populations. Fourth, the lncRNA H19 rs2839698 G>A, rs217727 G>A and rs2107425 C>T polymorphisms were analyzed separately, and the effects of haplotype and gene-gene interactions could not be analyzed with the available data. Fifth, heterogeneity existed in all three polymorphism loci. Clinical diversity (sometimes called clinical heterogeneity), methodological diversity (methodological heterogeneity) and statistical heterogeneity were the most common source. In our meta-analysis, many factors such as the diversity of cancer type, classification of disease severity, environment factors, might have increased heterogeneity and influenced our findings.

Despite these limitations, our meta-analysis indicated that the lncRNA lncRNA H19 rs2839698 G>A and rs2107425 C>T polymorphisms might play different role during cancer development. More studies in patients of different ethnicities and with larger number sizes are needed to confirm these findings.

## MATERIALS AND METHODS

### Search strategy

Two of the authors independently searched the online PubMed, Embase, and Science Citation Index (SCI) English databases for relevant studies published prior to December 1, 2016 that examined associations between H19 lncRNA polymorphisms and cancer risk. The bibliographies of relevant studies and recently reviews were retrospectively examined to identify additional articles. The following search terms and strategy were used:

#1 lncRNA H19

#2 long non-coding RNA H19

#3 H19

#4 rs2839698

#5 rs2107425

#6 rs217727

#7 #1 OR #2 OR #3 OR #4 OR #5 OR #6

#8 polymorphism

#9 variant

#10 mutation

#11 #8 OR #9 OR #10

#12 cancer

#13 tumor

#14 neoplasm

#15 #12 OR #13 OR #14

#16 #7 AND #11 AND #15

### Eligibility criteria

Studies were collected according to the following inclusion criteria: 1) case-control studies focused on the association between lncRNA H19 polymorphisms and cancer risk; 2) genotype distribution and frequency data provided were sufficient to estimate the odds ratios (ORs) and 95% confidence intervals (CIs); 3) studies published only in English; and 4) data for the largest sample set or most recent samples were included when data from the same set of patients were used more than once within a study.

### Data extraction and quality evaluation

Two investigators (Li and Yin) independently extracted the following relevant information from the included studies: abbreviated name of the first author, publishing date, country or region where the study was conducted, patient ethnicity (Asian/Caucasian), the design of the controls, sample sizes for patients and healthy controls, genotype distribution frequency data, genotyping method, Hardy-Weinberg equilibrium (HWE) for the controls, and cancer type. Quality evaluation of included studies was assessed by the first two authors using modified Newcastle-Ottawa scale (NOS), the scores ranged from 0 points (worst) to 10 points (best) (Table 3).

**Table 3:** Scale for quality evaluation

Criteria	Score
**Representativeness of cases**	
Consecutive/randomly selected form case population with clearly defined sampling frame	2
Consecutive/randomly selected form case population without clearly defined sampling frame or with extensive	1
Not described	0
**Source of controls**	
Population- or Healthy-based	2
Hospital-bases	1
Not described	0
**Hardy-Weinberg equilibrium in controls**	
Hardy-Weinberg equilibrium	2
Hardy-Weinberg disequilibrium	1
Not available	0
**Genotyping examination**	
Genotyping done under “blinded” condition and repeated again	2
Genotyping done under “blinded” condition or repeated again	1
Unblinded done or not mentioned and unrepeated	0
**Association assessment**	
Assess association between genotypes and cancer with appropriate statistics and adjustment for confounders	2
Assess association between genotypes and cancer with appropriate statistics and without adjustment for confounders	1
Inappropriate statistics used	0

### Statistical analysis

Crude ORs with 95% CIs were calculated to assess the strength between lncRNA H19 rs2839698 G>A, rs217727 G>A and rs2107425 C>T polymorphisms and cancer susceptibility. For the lncRNA H19 rs2839698 G>A polymorphism, the following five genetic models were used: allele contrast (A vs. G), co-dominant models (GA vs. GG and AA vs. GG), dominant model (GA+AA vs. GG), and recessive model (AA vs. GG+GA). Similar genetic models were also used to assess the lncRNA H19 rs217727 G>A and rs2107425 C>T polymorphisms. Stratified measurements were calculated based on ethnicity difference, HWE status, control design, and so on. Heterogeneity was calculated using Cochran's *Q* test and *I*^2^ statistic[41]. The fixed-effect model (the Mantel-Haenszel method) was adopted when the I^2^ value less than 50%[42]. Otherwise, a random-effects model (DerSimonian and Laird method) was applied [43, 44]. Meta-regression was conducted to examine analyses that exhibited heterogeneity. Cumulative meta-analyses were conducted to determine whether the results changed significantly as the number of studies included increased. Furthermore, sensitivity analyses were also performed to examine the stability of the results when studies were removed one at a time. Both Egger's linear regression and Begg's funnel plots were used to assess the potential publication bias [45, 46]. All statistical calculations were performed with STATA version 14.0 (Stata Corporation, College Station, TX, USA). A *P* value < 0.05 was considered statistically significant.

## SUPPLEMENTARY MATERIALS FIGURES AND TABLES


