# Supplementary material for: Significant association between lncRNA H19 polymorphisms and cancer susceptibility: a meta-analysis
Source: Oncotarget. 2017 Mar 29;8(28):45143–53. doi: 10.18632/oncotarget.16658 (PMC5542173; doi:10.18632/oncotarget.16658)
Supplement: Supplementary file 1 [file oncotarget-08-45143-s001.pdf]

Significant association between lncRNA H19 polymorphisms and cancer susceptibility: a meta-analysis

Supplementary Materials

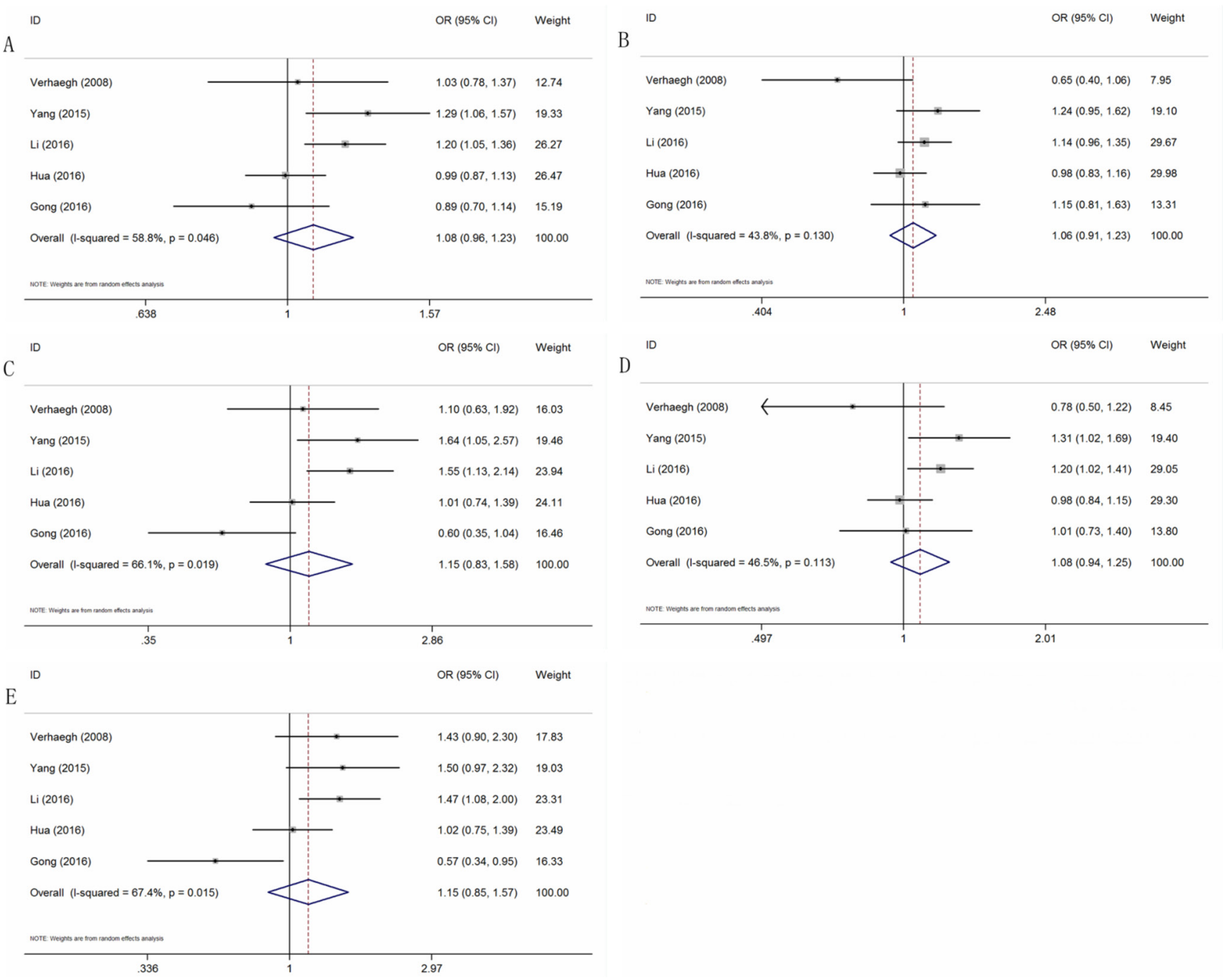

Supplementary Figure 1: OR and 95% CIs of the associations between rs2839698 G>A polymorphism and cancer risk. (**A**) for A vs. G model; (**B**) for GA vs. GG model; (**C**) for AA vs. GG model; (**D**) for GA+AA vs. GG model; (**E**) for AA vs. GG+GA model).

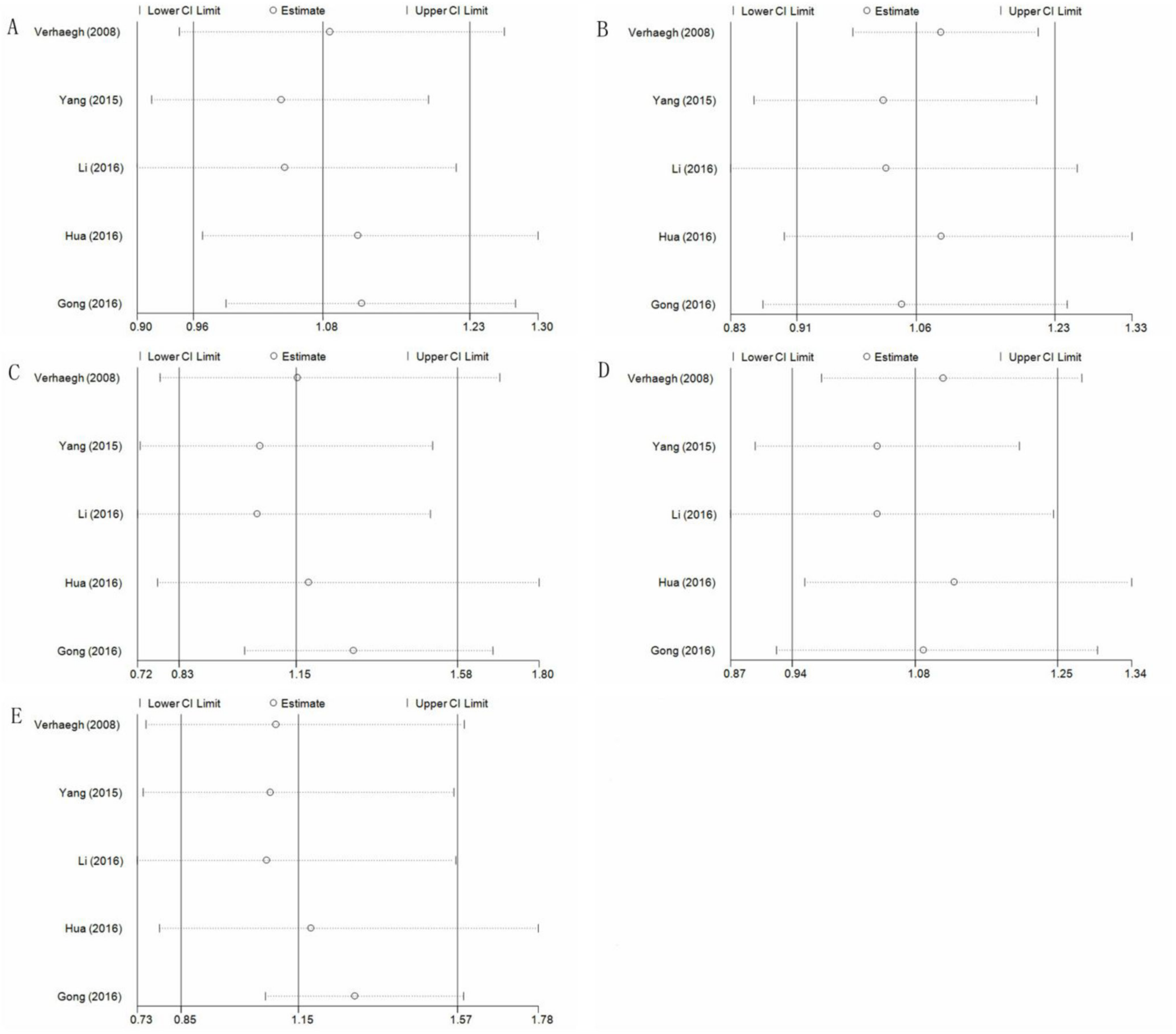

Supplementary Figure 2: Sensitivity analyses according to publication year in rs2839698 G>A polymorphism. (**A**) for A vs. G model; (**B**) for GA vs. GG model; (**C**) for AA vs. GG model; (**D**) for GA+AA vs. GG model; (**E**) for AA vs. GG+GA model).

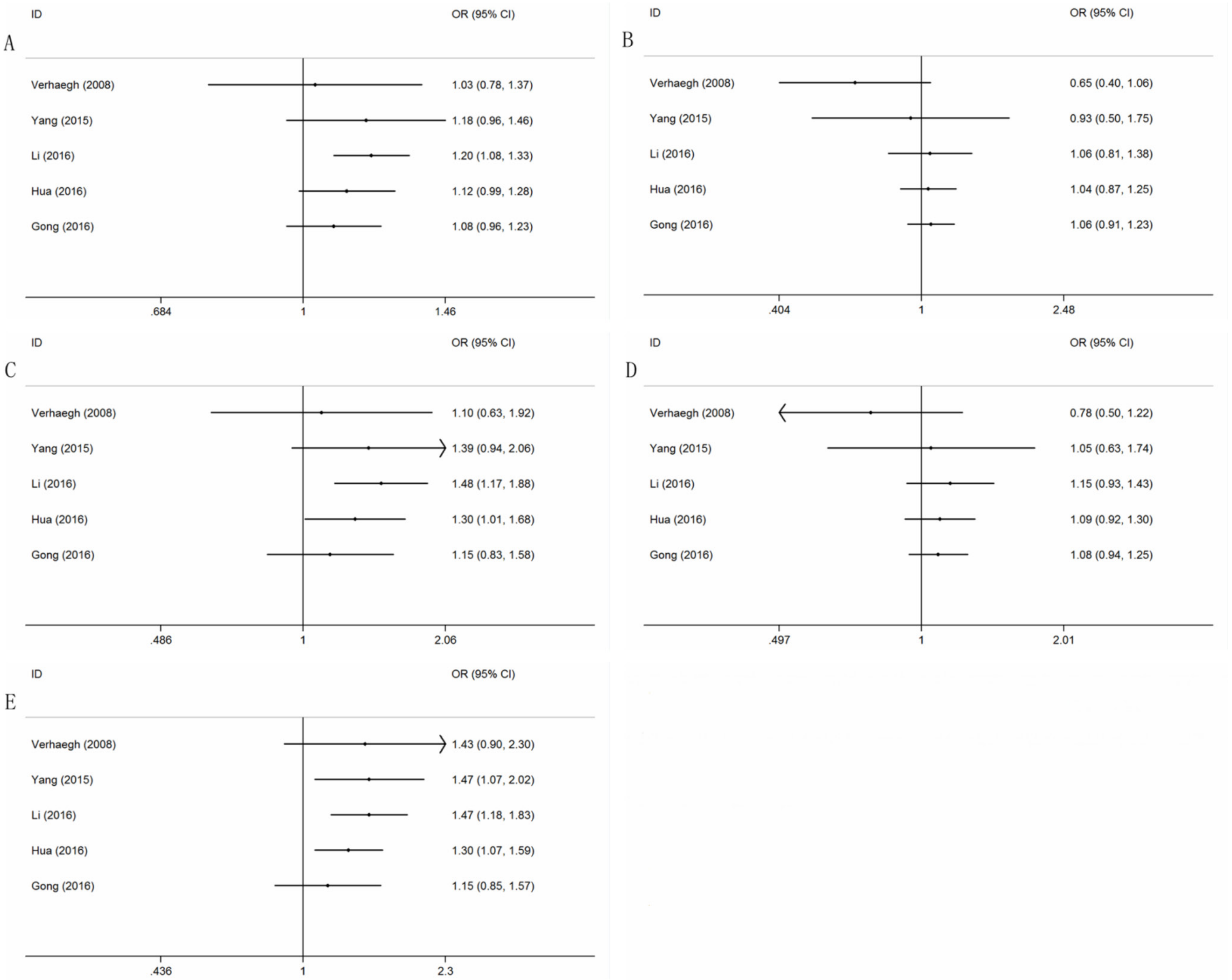

Supplementary Figure 3: Cumulative meta-analyses according to publication year in rs2839698 G>A polymorphism. (**A**) for A vs. G model; (**B**) for GA vs. GG model; (**C**) for AA vs. GG model; (**D**) for GA+AA vs. GG model; (**E**) for AA vs. GG+GA model).

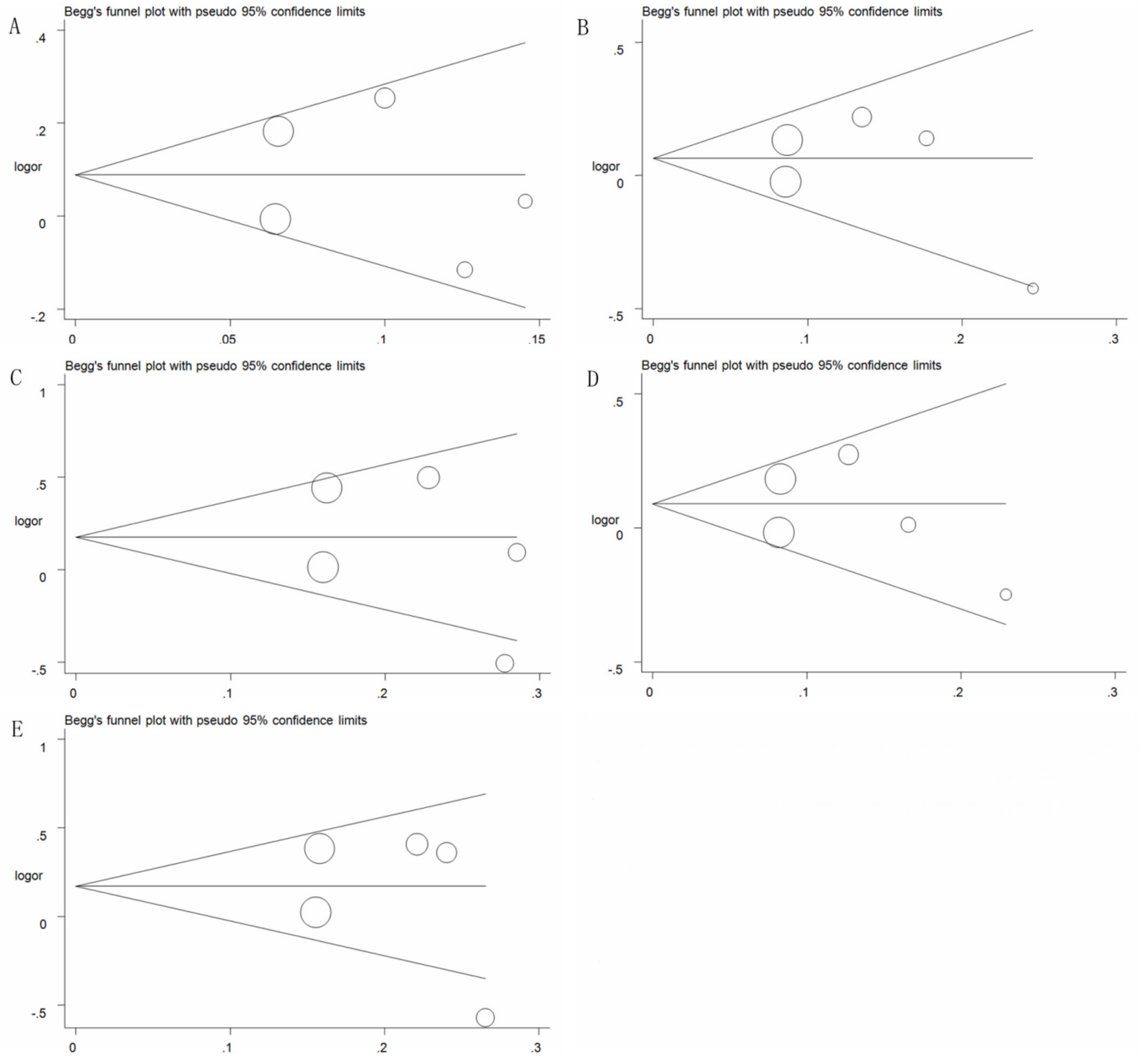

Supplementary Figure 4: Funnel plot analysis to detect publication bias in rs2839698 G>A polymorphism. (**A**) for A vs. G model; (**B**) for GA vs. GG model; (**C**) for AA vs. GG model; (**D**) for GA+AA vs. GG model; (**E**) for AA vs. GG+GA model). Circles represent the weight of the studies.

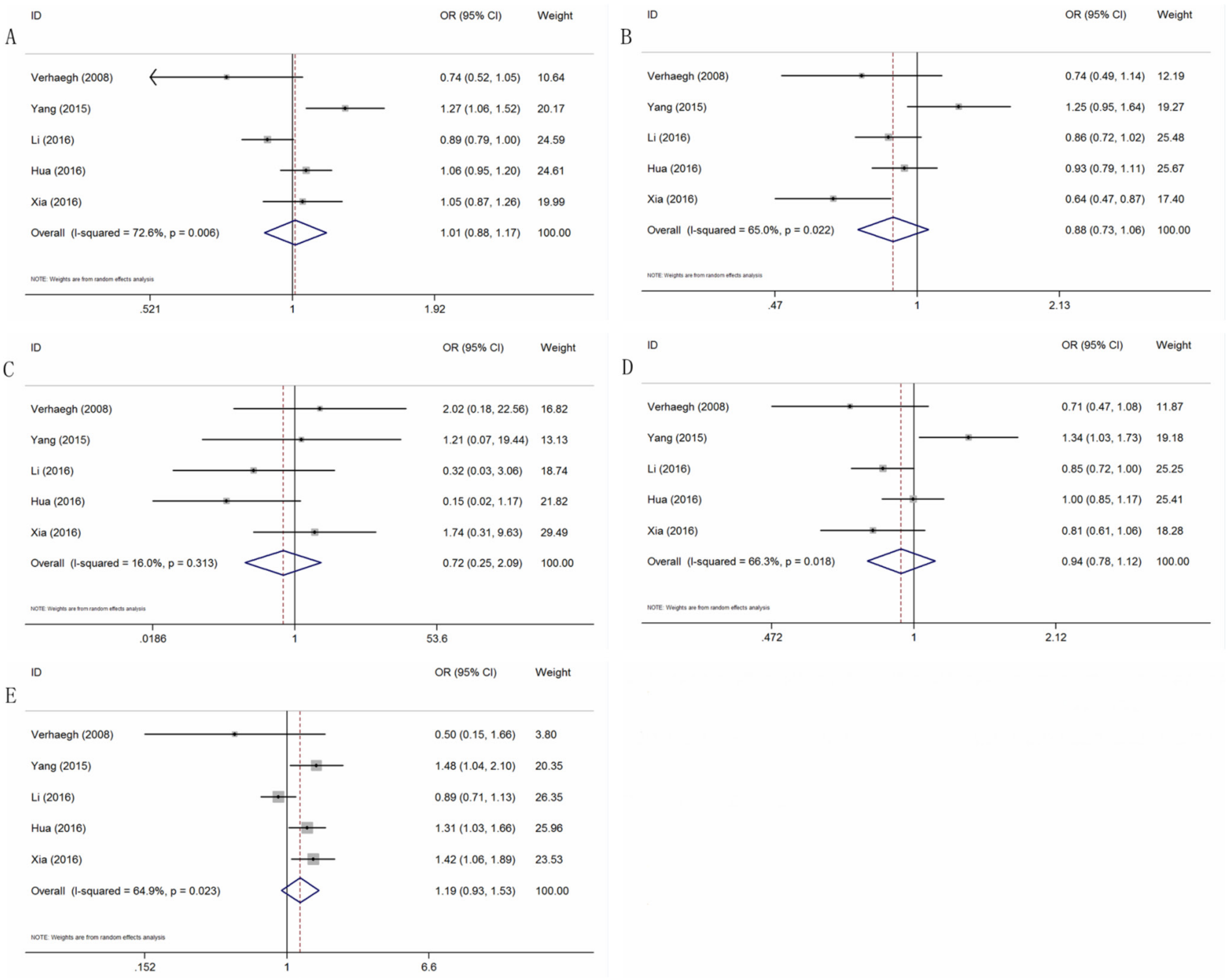

Supplementary Figure 5: OR and 95% CIs of the associations between rs217727 G>A polymorphism and cancer risk. (**A**) for A vs. G model; (**B**) for GA vs. GG model; (**C**) for AA vs. GG model; (**D**) for GA+AA vs. GG model; (**E**) for AA vs. GG+GA model).

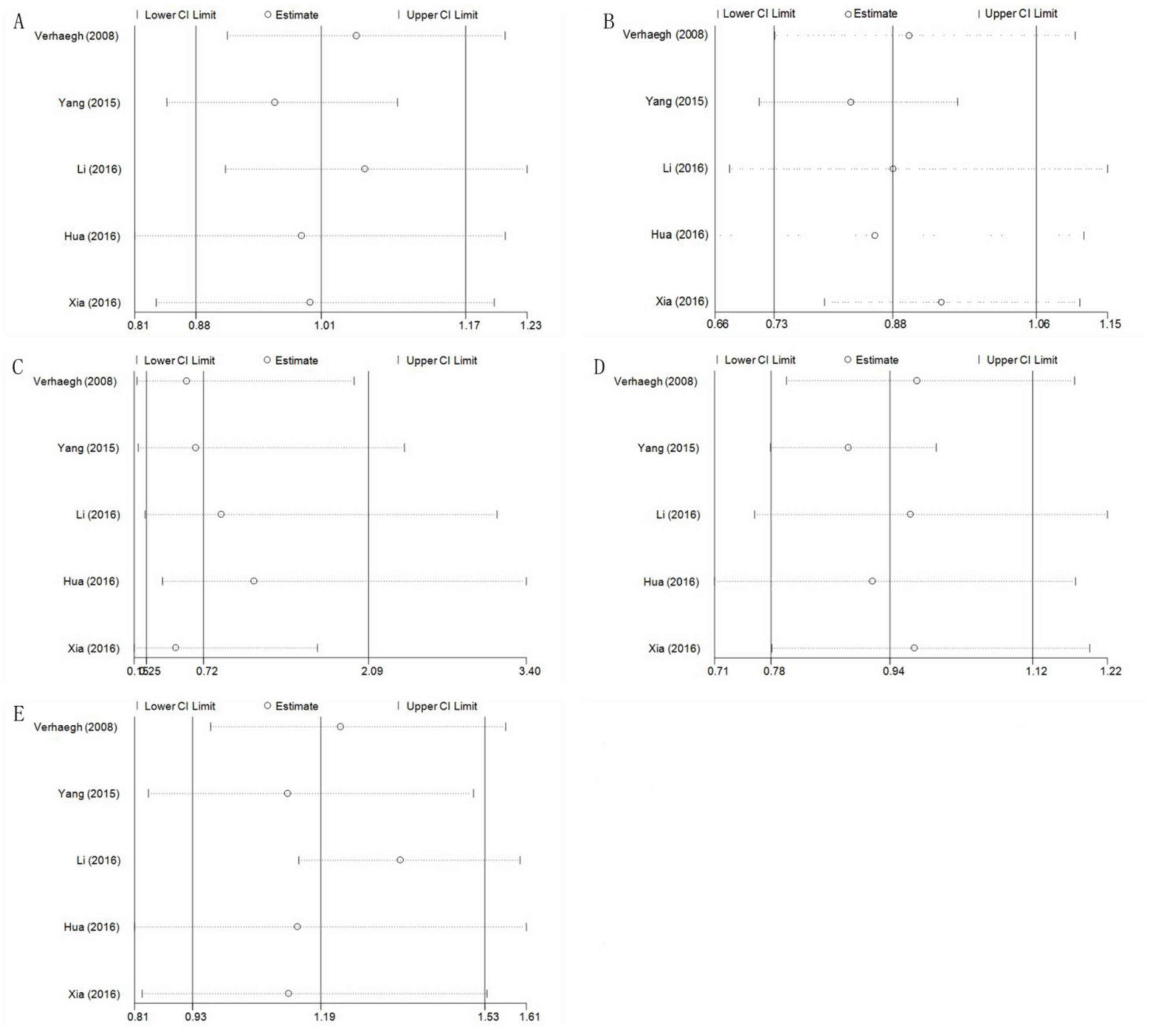

Supplementary Figure 6: Sensitivity analyses according to publication year in rs217727 G>A polymorphism. (**A**) for A vs. G model; (**B**) for GA vs. GG model; (**C**) for AA vs. GG model; (**D**) for GA+AA vs. GG model; (**E**) for AA vs. GG+GA model).

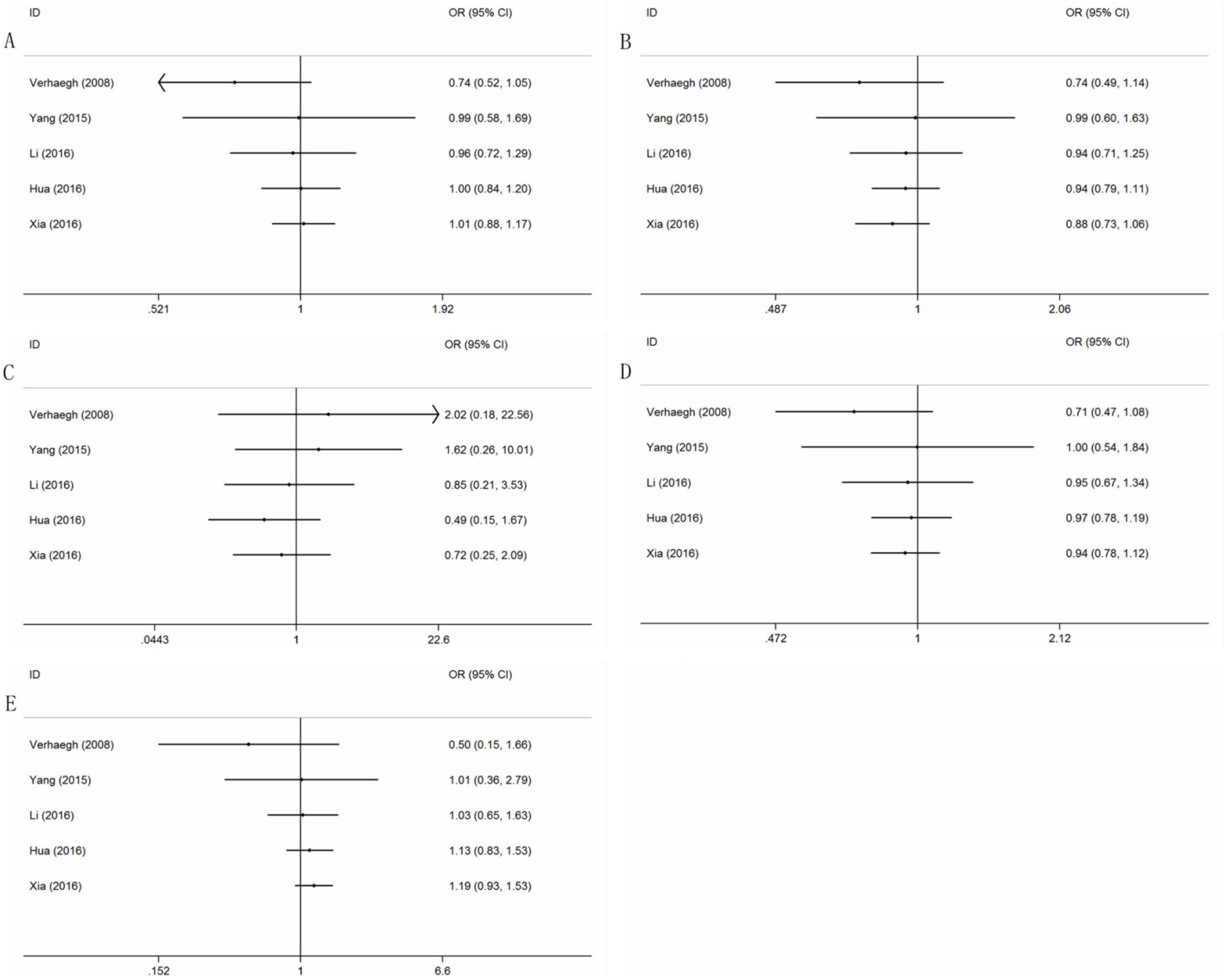

Supplementary Figure 7: Cumulative meta-analyses according to publication year in rs217727 G>A polymorphism. (**A**) for A vs. G model; (**B**) for GA vs. GG model; (**C**) for AA vs. GG model; (**D**) for GA+AA vs. GG model; (**E**) for AA vs. GG+GA model).

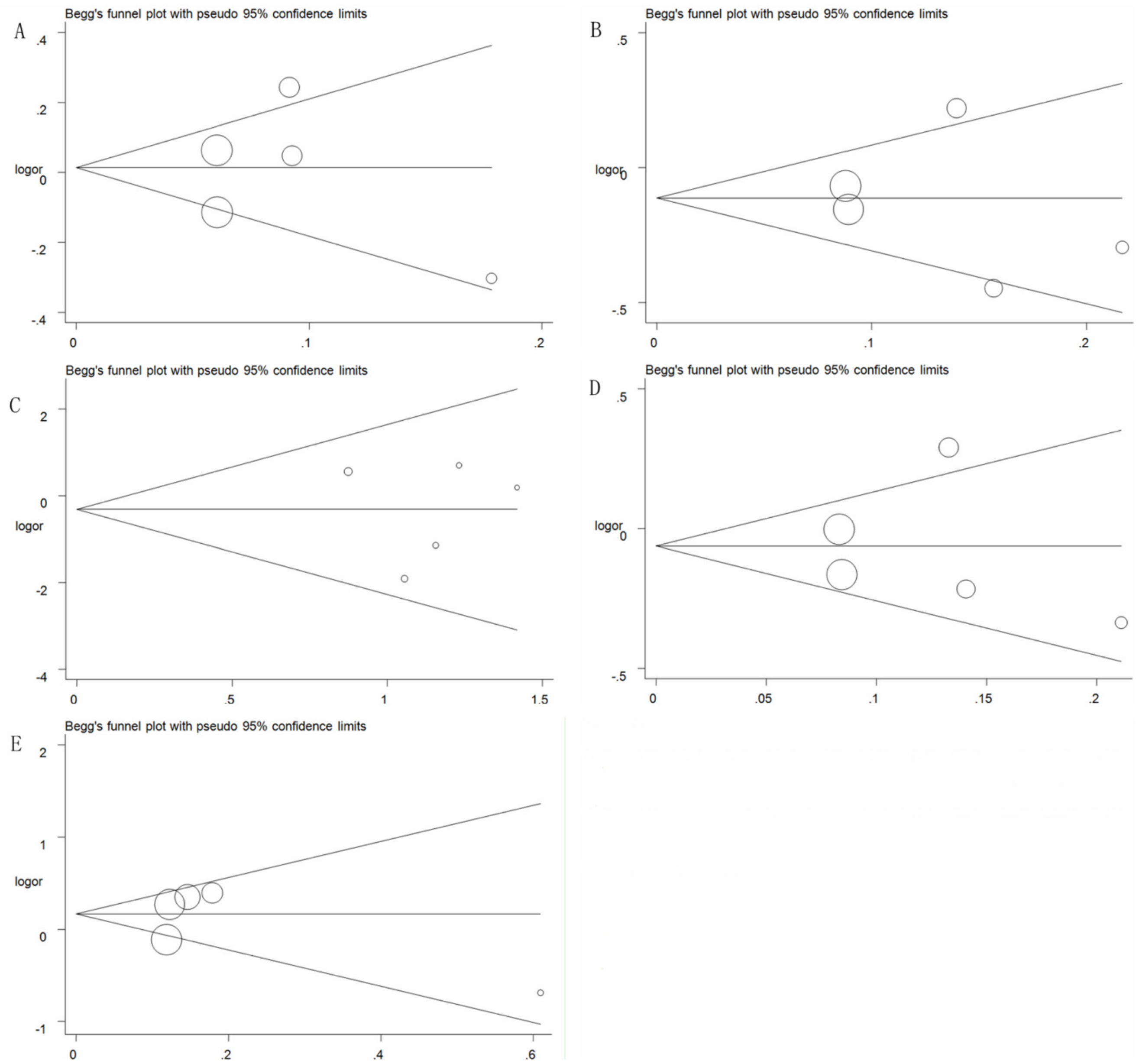

Supplementary Figure 8: Funnel plot analysis to detect publication bias in rs217727 G>A polymorphism. (**A**) for A vs. G model; (**B**) for GA vs. GG model; (**C**) for AA vs. GG model; (**D**) for GA+AA vs. GG model; (**E**) for AA vs. GG+GA model). Circles represent the weight of the studies.

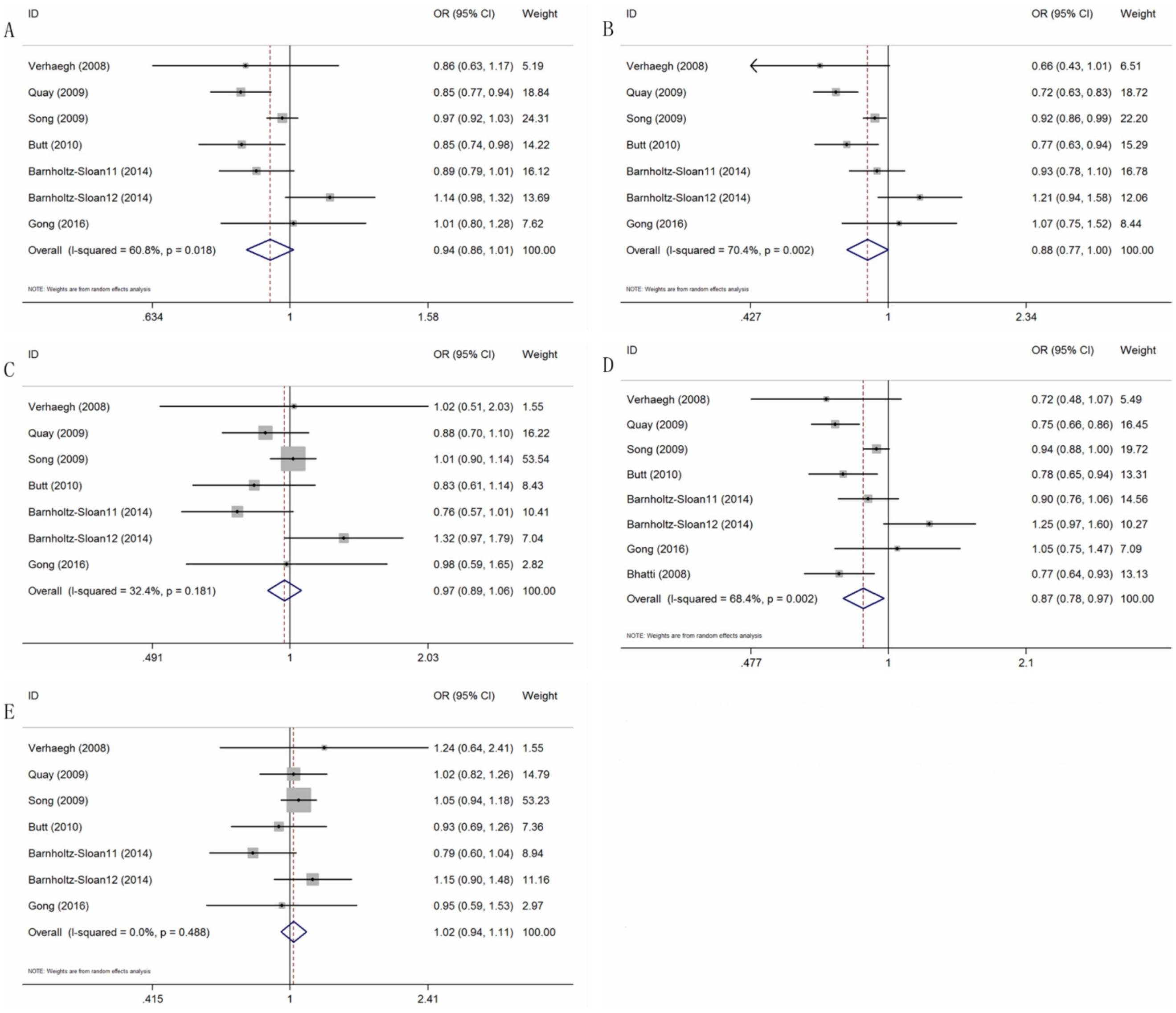

Supplementary Figure 9: OR and 95% CIs of the associations between rs2107425 C>T polymorphism and cancer risk. (**A**) for T vs. C model; (**B**) for CT vs. CC model; (**C**) for TT vs. CC model; (**D**) for CT+TT vs. CC model; (**E**) for TT vs. CC+CT model).

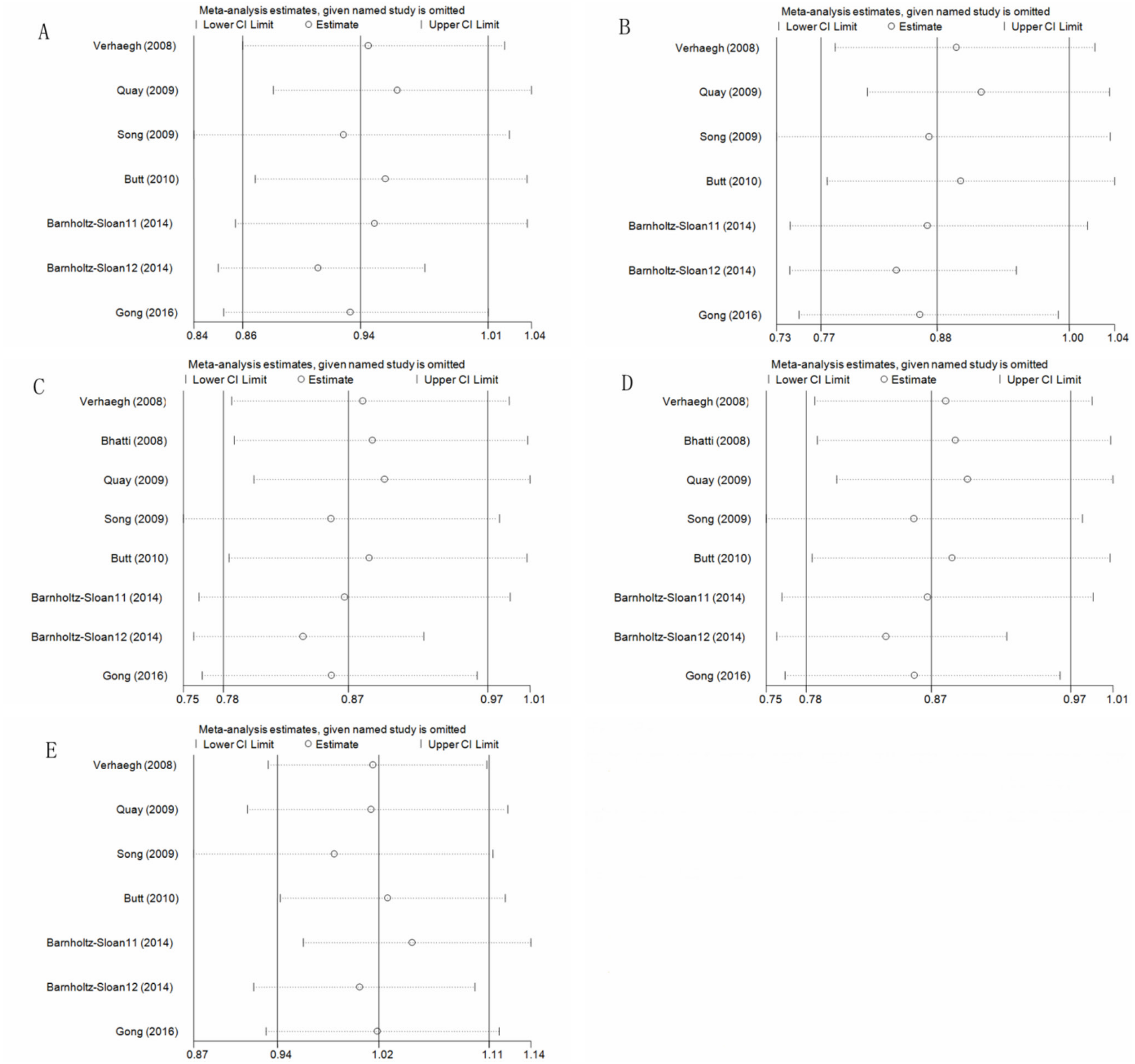

Supplementary Figure 10: Sensitivity analyses according to publication year in rs2107425 C>T polymorphism. (**A**) for T vs. C model; (**B**) for CT vs. CC model; (**C**) for TT vs. CC model; (**D**) for CT+TT vs. CC model; (**E**) for TT vs. CC+CT model).

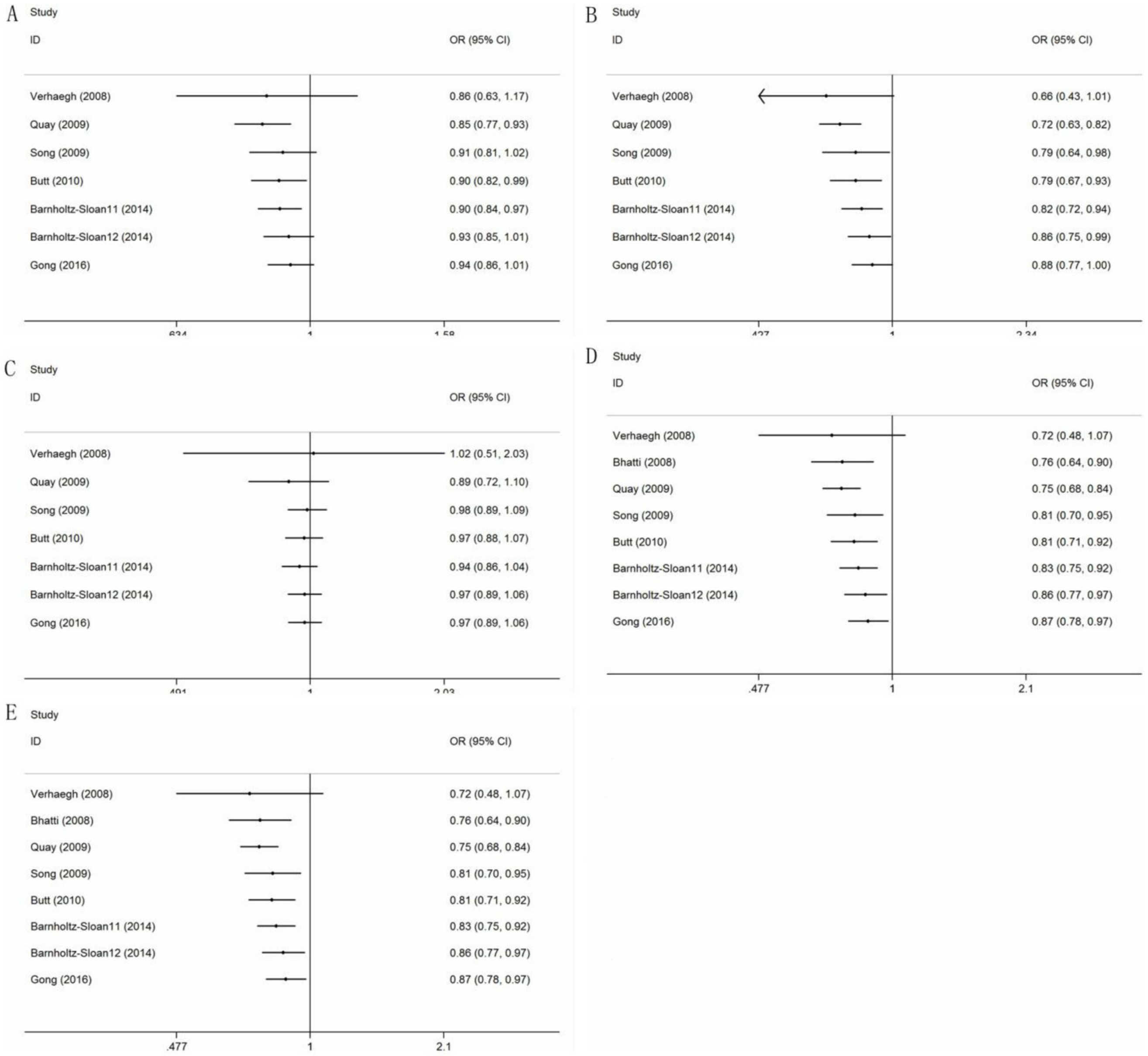

Supplementary Figure 11: Cumulative meta-analyses according to publication year in rs2107425 C>T polymorphism. (**A**) for T vs. C model; (**B**) for CT vs. CC model; (**C**) for TT vs. CC model; (**D**) for CT+TT vs. CC model; (**E**) for TT vs. CC+CT model).

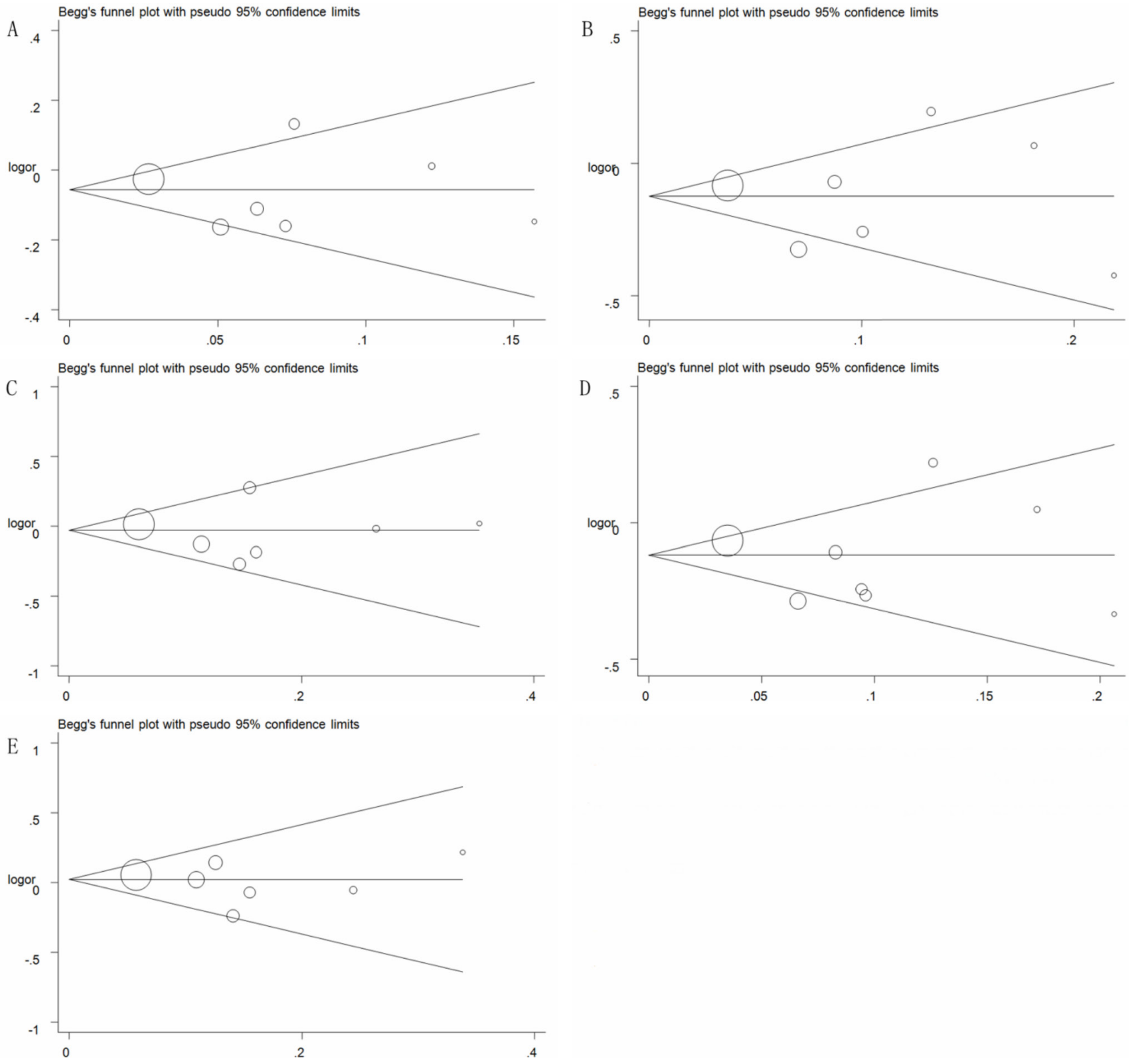

Supplementary Figure 12: Funnel plot analysis to detect publication bias in rs2107425 C>T polymorphism. (**A**) for T vs. C model; (**B**) for CT vs. CC model; (**C**) for TT vs. CC model; (**D**) for CT+TT vs. CC model; (**E**) for TT vs. CC+CT model). Circles represent the weight of the studies.
